# Effect of Cell Morphology on Flexural Behavior of Injection-Molded Microcellular Polycarbonate

**DOI:** 10.3390/ma15103634

**Published:** 2022-05-19

**Authors:** Kübra Güzel, Jan-Christoph Zarges, Hans-Peter Heim

**Affiliations:** Institute of Material Engineering, Polymer Engineering, University of Kassel, 34125 Kassel, Germany; zarges@uni-kassel.de (J.-C.Z.); heim@uni-kassel.de (H.-P.H.)

**Keywords:** polycarbonate, cell morphology, flexural properties, subset regression analysis, IR-thermography

## Abstract

The quantitative study of the structure and properties relationship in cellular materials is mostly limited to cell diameter, cell density, skin layer thickness, and cell size distribution. In addition, the investigation of the morphology is generally carried out in two dimensions. Therefore, the interrelation between morphological properties and mechanical characteristics of the foam structure has remained in an uncertain state. In this study, during the physical foaming process, a foam morphology is locally created by using a mold equipped with a core-back insert. The variation in morphology is obtained by modifying the mold temperature, injection flow rate, and blowing agent content in the polymer melt. X-ray microtomography (μCT) is used to acquire the 3D visualization of the cells structure. The Cell Distribution Index (*CDI*) is calculated to represent the polydispersity in cell size distribution. The relationship between the wide range of morphological qualities and relevant flexural properties is made explicit via a statistical model. According to the results, the morphology, particularly cell shape, characterizes the mechanism of the linear elastic deformation of the closed-cell foams. IR-thermography reveals the bending failure of cellular structures in the tensile region despite the differences in cell diameter.

## 1. Introduction

Foam injection molding (FIM) of thermoplastic polymers is well-recognized due to several processing advantages. For instance, the melt viscosity reduction leads to a decrease in the required injection pressure. The creation of a uniform internal holding pressure due to the melt expansion reduces the necessity of applying holding pressure as well as the magnitude of clamping forces. In addition, the lessened amount of component weight ensures a shortened cooling time, in the case of an achieved high stability of internal pressure. Apart from process-related advantages, foam injection molding provides more freedom of product design on a vast scale compared to conventional injection molding by avoiding shrinkage [1,2,3,4,5].

In the conventional microcellular injection molding known as low-pressure FIM, the foaming area is bordered by a stationary mold cavity. In this case, cell nucleation and growth start upon the pressure drop during the mold filling stage. Along the foaming process, blowing agent pressure is the determinant of the cavity pressure. This leads to pressure differences along the flow path, restricted density reduction, and hindered uniform cell growth [6]. In most cases, the achieved density reduction is less than 15% [5,7,8]. In order to attain a higher density reduction as well as a desirable uniform cellular structure, the high-pressure-FIM process is combined with a breathing mold (core-back) technology. In this method, the cavity volume is filled with a single-phase melt/gas and is subsequently enlarged in one direction by core pulling or the retraction of the clamping unit. The high cavity pressure induces the cells that had nucleated during the mold filling stage to resolve into the melt before the cavity expansion. However, the final foam structure is affected by FIM processing conditions. Therefore, it is essential to understand the influence of processing parameters on achieving a uniform final structure with the required properties. Shaayegan et al. [9] examined the effect of the injection speed, injection gate resistance and blowing agent content, melt flow index, and nucleating agent on the bubble formation and growth in the high-pressure FIM by using an on-line visualization method. It was found that the cell density is increased with the concentration of the dissolved blowing agent. Tromm et al. [10] investigated the evolution of the cells during the mold-filling stage in the high-pressure injection molding process with a mold opening system. A higher packing pressure and longer packing time was found to be influential parameters for the deformation of cells and reduction in the cell size before the mold opening. The entire resolution of these premature cells and packing process results in a more uniform cellular structure after mold opening. Consequently, a flow path-independent, uniform cellular structure and higher density reduction can be achieved [10]. Spörrer and Altstädt evaluated the cell morphology produced in physical foam injection molding on a core-back mold technology as a function of processing parameters. According to the results, the preheated mold leads to a broadened cell size distribution. A thinner compact skin layer is obtained by lowering the thermal gradient between the melt and mold surface [4]. Lee et al. achieved a uniform cell structure with a void fraction over 40%. To that end, an advanced mold opening technology was used. Gas content, melt temperature, and injection flow rate were varied to examine the effects on the void fraction, the degree of mold filling, and cell size uniformity. It was found that the higher the blowing agent content is, the more uniform the void fraction is throughout the final part’s morphology. A smaller cell size with a narrow cell size distribution is obtained as the injection flow rate is increased to 160.8 mL/s [11]. The type of blowing agent is also effectual on the resulting product properties. For instance, the use of a chemical blowing agent causes diminished thermal stability, whereas a physical blowing agent results in a smaller cell size and higher cell density. It was also found to be more suitable for achieving low-density foams [3,12,13,14,15,16].

Microcellular foam injection molding promises these aforementioned process and product design advantages. However, the physical properties of the cell structure such as cell size, cell size distribution, wall thickness, and foam density play important roles in the mechanical performance [16,17,18,19,20,21,22]. To avoid the deterioration in material properties, it is crucial to understand the interrelationship between the morphology and mechanical properties. Voiconi et al. investigated the microstructure of foam materials and determined the flexural properties of rigid PUR foams by using a Digital Image Correlation (DIC) technology. The results showed that the main mechanical properties such as flexural modulus and flexural strength increase with the density [20]. Bledzki et al. pointed out the relationship between morphological properties and mechanical properties. Density was found to be the most substantial parameter to the describe mechanical characteristics of the foam morphologies [16]. Bledzki et al. also examined the breaking behavior of microcellular foamed polycarbonate depending upon cell diameter, cell distance, and also compact skin layer thickness by applying the Charpy notched impact test. It was found that a sandwich structure creation with a thick compact surface layer, high density, large cell distances, and small cell sizes in the center area has a positive effect on the notched impact strength [17]. Güzel and Heim [21] determined the relationship of processing parameters such as blowing agent content, injection flow rate, and mold temperature to the consequential properties of microcellular PC. The results showed that a finer cellular structure produces a higher amount of plastic deformation than a smaller cell structure and also the reduction in cell size leads to an increment in tensile strength. When it comes to tensile strength, it was found to be proportional to foam densities [22].

Skin layer thickness is defined as a compact, unfoamed layer in the foamed component. As the gas cells close to the mold surface can diffuse out from the material, cell nucleation can be delimitated toward the component surface, which results in a sandwich structure [23]. Skin layer thickness can also influence mechanical properties by changing the amount of material per unit area. An incomplete skin layer results in a considerable decrease in mechanical properties [15]. Wong et al. achieved a 10% increase in tensile properties by increasing the skin layer thickness from 100 to 500 µm [24]. Spörrer and Altstädt found out that thicker skin layers result in an increase in flexural modulus [4].

Most morphological studies have been limited to quantitative analyses of cell size, cell density, cell size distribution, and surface layer thickness. The examination of morphology has generally been carried out by scanning electron microscopy (SEM), due to its simplicity of use, economy, and less time needed in comparison to X-ray microtomography (µCT). However, images are influenced by the angle of the fractured surface and shape of the cell. X-ray microtomography can be used to reach realistic information in 3D space such as roundness of cells and cell volume distribution [10,21,25]. Although there are many qualitative analyses of cell size distribution, the correlation between the cell size distribution and mechanical properties is ambiguous. The Cell Distribution Index (*CDI*) is a quantitative parameter for the characterization of polydispersity in cell size distribution. The *CDI*-value close to unity indicates the uniformity in cell size, and it can be used to quantify the cell size distribution [26,27].

In this study, the processing parameters, such as mold temperature, blowing agent concentration, and volume flow rate, are varied to create different morphological properties (cell size, distance between cells, cell density, and skin layer thickness). X-ray micro-computer tomography (μCT) is carried out to obtain the 3D visualization of the cells structure. In addition, the sphericity of cells and cell volume are also obtained to investigate the interrelationship between morphological properties and mechanical properties. The polydispersity in cell size is also represented by *CDI* as the homogeneity of cell size distribution. On the basis of these morphological analyses and the results of bending tests, correlations are established among structural properties and flexural properties. Within the scope of the study, multiple regression is used to rank which structural features are most important to predict flexural properties. Cell diameter, cell distance, compact layer thickness, cell density, sphericity, homogeneity of the cell size distribution, density reduction, and cell volume are quantitatively identified as structural explanatory variables. Consequently, equations are created based on the appointed model between the structural explanatory variables and the corresponding flexural properties of physically foamed polycarbonate.

## 2. Materials and Methods

The analyzed material was pure polycarbonate (Makrolon^®^ 2405) provided by Covestro AG (Leverkusen, Germany) with a melt volume rate (MVR) of 19 cm^3^/10 min (300 °C 1, 2 kg) and a density (ρ) of 1.20 g/cm³.

The molded part with dimensions of 120 mm × 80 mm and the initial part thickness of 3 mm was produced by using an all-hydraulic single-component injection molding machine Arburg Allrounder 470S (Arburg GmbH + Co KG, Loßburg, Germany) equipped with a shut-off nozzle of the hot-runner system and an injection unit with a 25 mm MuCell-screw.

### 2.1. Foam Injection Molding

**Figure 1:**
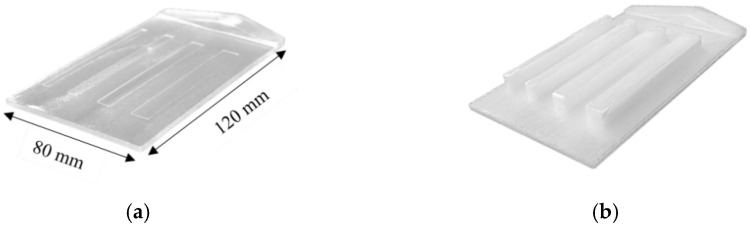
(**a**) Initial part thickness of 3 mm; (**b**) the extended ribs height 8.5 mm after cavity expansion.

Throughout the experiments, the polymer was processed at a melt temperature of 290 °C with a precise amount (given in Table 1) of supercritical nitrogen (N_2_), which was introduced as a physical blowing agent into the molten polymer by using MuCell^®^ technology (Trexel Inc., Woburn, Massachusetts). The mold was also equipped with a core-back insert to mold four rectangular ribs of different widths (4/6/8/10 mm) (shown in Figure 1). After the implementation of 60 MPa of packing pressure for 2 s and subsequently 3 s of delay time (t_D_, time between volumetrically filling the initial core cavity and expansion of the core), the pressure drop was induced by the core movement. Consequently, the height of the ribs was adjusted from 0.5 to 8.5 mm in addition to the 3 mm thickness of the plate. At the final thickness of the foamed part, 45 s of cooling time was applied prior to ejection. The mold temperature, injection flow rate, and blowing agent content in the polymer melt (SCF-level, supercritical fluid level) were varied to achieve variance in foam morphologies (Table 1).

### 2.2. Mechanical Characterization

**Figure 2:**
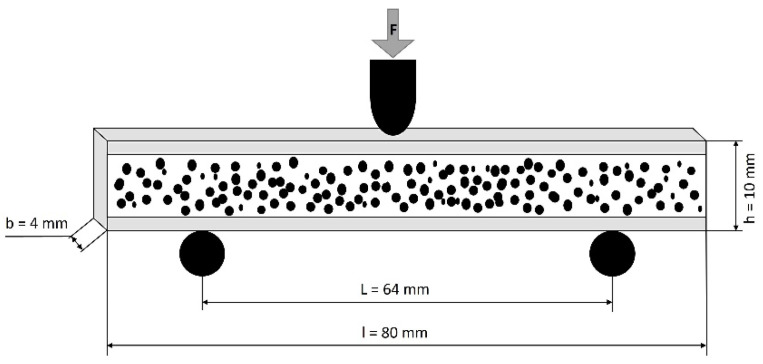
Detailed test specimen position for three-point bending test (“h” thickness, “b” width and “l” length ofthe test sample, “L” is the support span length, “F” is the applied force).

The extended rib with a width of 10 mm was tested on a universal testing machine Z010 (Zwick Roell, Ulm, Germany) with a constant crosshead speed of 2 mm/min at standard room temperature and relative humidity (25 °C, 50%). Test specimens were concentrically milled out of the produced components by a Computerized Numerical Control (CNC) machine (DMO 100 Monoblock, Deckel Maho, Bielefeld, Germany) to test the foamed components. The compact skin layer on the lateral sides of the specimens (Figure 2) were not removed, to determine the influence of the compact skin layer thickness on the bending properties of foamed PC.

### 2.3. Morphology Characterization

The morphologies of selected molded specimens were examined using a digital light microscope (Keyence VHX series, Keyence, Ōsaka, Japan) with the dark-field method (dark cells and bright matrix, 50× magnification).

**Figure 3:**
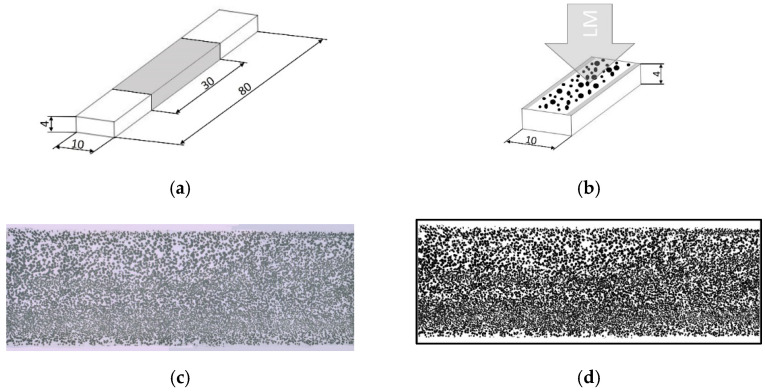
An illustration of the morphological examination process: (**a**) the elaboration of the cut area, (**b**) investigation area of digital light microscopy, (**c**) microscopic image of the representative specimen, and (**d**) the microscopic image after image processing by applying the “Threshold” (“LM” denotes light microscopy).

The rib with the width of 10 mm was removed from the compact part (Figure 1a) and cut in the middle with the length of 30 mm (shown in Figure 3a). These cuts with skin layers were embedded in resin, grinded, and polished in order to measure cell diameter, distance between cells, and thickness of the compact skin layer (Figure 3b). Furthermore, the microscopic images (Figure 3c) were subjected to a threshold with the help of a public domain image processing software (ImageJ). Threshold is a command that separates the cells of interest (colored black) from the compact material (colored white) in the morphology (Figure 3d). Hereby, the frequency of cells as well as the area of cells were computed. Accordingly, cell density, frequency, and CDI were calculated as indicators of the foam structure. At least 3 samples were examined for each morphological indicator.

The cell density (N) is the number of cells created per unit volume (cm^3^). It was determined using the following equation [28]:

(1) $$N={\left(\frac{n}{A}\right)}^{\frac{3}{2}}\times \frac{\rho_{s}}{\rho_{F}}\;$$

where *n* is the number of cells counted in the digital light microscope image, *A* is the area of the microscopic image (cm^2^), and $\rho_{s}$ and $\rho_{F}$ indicate the density of the solid and foam material (g/cm^3^), respectively.

In the literature, the average cell size was mostly used to understand the mechanical behavior of foamed material. However, *CDI* is an important parameter to define foam morphology because it quantifies the deviation in cell size as a measure of homogeneity and enlightens the cell distribution pattern. Therefore, *CDI* was calculated by using the following Equation (2) proposed by Rizvi et al. [26]. A value of 1 represents monodispersion where the cells size is equal and the variation in cell size leads to an increase in *CDI*.

(2) $$CDI=\frac{\Phi_{D}}{\Phi_{N}}\;$$

(3) $$\Phi_{N}=\frac{\sum_{i}N_{i}\Phi_{i}}{\sum_{i}N_{i}}\;$$

(4) $$\Phi_{D}=\frac{\sum_{i}N_{i}\Phi_{i}^{2}}{\sum_{i}N_{i}\Phi_{i}}\;$$

where ($\Phi_{i}$) and ($N_{i}$) indicate the diameter of cells in microns and the number of the cells having diameter $\Phi_{i}$, respectively.

A density measurement was carried out with a density measuring system of the type 6060/60801 from Sartorius AG (Göttingen, Germany). This system is equipped with a precision measuring scale and a fixture for determining the sample weight in a reference liquid based on the buoyancy method. Water (density ~1 g/cm³) was used as a reference liquid for all measurements. A piece of 20 mm in length, 10 mm in width, and 4 mm in height with the compact skin layer cut from the middle of the rib was used. Three samples were measured for each test run.

Three-dimensional X-ray microtomography (μCT) was performed using a Zeiss XRadia 520 Versa microscope (Zeiss, Oberkochen, Germany). The re-constructed data were assessed by the help of AVIZO (FEI, Hillsboro, OR, USA) software. Rectangular samples ~20 mm in length, ~10 mm in width, and ~3 mm in height were scanned to depict the sphericity of cells and the cell volume. For each sample, 1601 images were taken with a resolution of 2.07 μm/pixel and exposure time of 3.5 s with a standard voltage setting of 80 kV and 7 W.

All the information that was obtained from the mentioned investigation methods culminated into the elaboration of the relationship between the wide range of morphological qualities and relevant flexural properties. Hereby, developing a statistical model to predict the mechanical responses is in the scope of this study.

Simple linear regression is practiced to examine the relationship between a quantitative response variable and single quantitative explanatory variable. Multiple linear regression is an extension of single linear regression and used in the case of more than one independent variable that predicts or explains the response variable (shown in Equation (5)).

(5) $$Y=\beta_{0}+\beta_{1}X_{1}+\beta_{2}X_{2}+\ldots +\beta_{m}X_{m}+e\;$$

where {*X_1_,X_2_…X_m_*} *m* is the number of independent variables with *n* observation responses {*Y*} in the presence of errors $\left\{e\right\}$. {*β*_1_} is the y-intercept (constant term) and {*β*_1_, *β*_2_…*β*_k_} are the coefficients for each explanatory variable [29]. To build a multiple regression model, it is crucial to know how many and which variables fit in the model. Using all present variables to create a model generates several data analysis problems [28,30]. For instance, irrelevant variables micrify the significant relationship that exists between other variables. In addition, the number of investigations carried out on each variable should be higher than the number of variables.

Best subset regression is an efficient way to select variables with respect to statistical significance prior to the modeling process [31]. It is used to compare possible regression models that $2^{m}$ submodels are created within the subset of the identified explanatory variables. These models are presented as two best models for one predictor, two predictors, and three predictors until the whole set of predictors is used in one model. In this study, flexural strength and flexural modulus were chosen as response variables, and cell density, cell diameter (median), cell distance (average), cell volume (median), compact layer thickness, density reduction, homogeneity of the cell size distribution, and sphericity were identified as explanatory variables. To distinguish the best subset, the model fit statistics are compared. The R^2^-value and adjusted R^2^-value (R^2^_adj_) are the coefficients of determination and used to measure the predictability of flexural properties. The S-value (square root of MSE) is the standard deviation of the error in the model, which represents the distance of the measured values falling from the fitted values. The lower the value of S, the better the model estimates the response. The predicted R^2^-value is also taken into account to determine how well the model predicts the response of new observations [32]. A statistical program named Minitab (State College, PA, USA) was used to compare different regression models that contain subsets of variables as well as attain the model fit statistics. Accordingly, the best fitting model with higher R^2^-value was determined with as few variables as possible.

## 3. Results and Discussion

**Figure 4:**
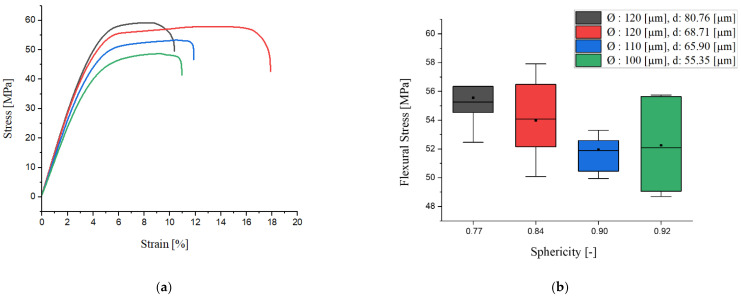
(**a**) Flexural stress and strain diagram of specimens; (**b**) flexural stress of distinctive cell morphologies (Ø: cell diameter, d: cell distance).

Figure 1a illustrates the stress–strain curves of the specimens that are perpendicularly loaded to the rise direction of the foam structure. In the figure, the specimen colored red has a 120 μm cell diameter and cells are placed at around 68 μm of distance. The black-colored specimen acquires, likewise, a 120 μm cell diameter. However, the distance between cells is larger (around 80 μm). The blue-colored specimen is dominated by the cell size of 110 μm with around 65 μm of distance between them. Lastly, the green-colored specimen has the smallest cell diameter of 100 μm and cell distance of 55 μm. It can be seen that in Figure 4a, a higher stress is necessary to propagate a crack with a larger cell structure and separation between cells. In addition, the strain at failure shows a proportional increment with the distance between cells up to approx. 69 μm. Figure 4b shows the box plots of flexural stress of the relevant specimens depending on the sphericity of the cells. Hereat, a sphericity of 1 [-] means perfect roundness. The morphology, particularly cell shape, characterizes the mechanism of the linear elastic deformation of the closed-cell foams. The relatively isotropic shape of cells leads to an attenuation in material stiffness and less strain at failure. However, Huber and Gibson [33] deduced that the anisotropy of the cell structure influences the modulus and collapse stress more than fracture toughness. During the plastic deformation process, buckling is found to be the dominant deformation mode and is followed by a small amount of linear hardening [34]. In Figure 4b, the standard deviation of the flexural strength results depending upon the sphericity of the cells show an indefinite relationship. It can be concluded that the flexural strength is concurrently influenced by several morphological properties. However, the median and the mean value of flexural strength show a linear decrease with an increment in sphericity.

**Figure 5:**
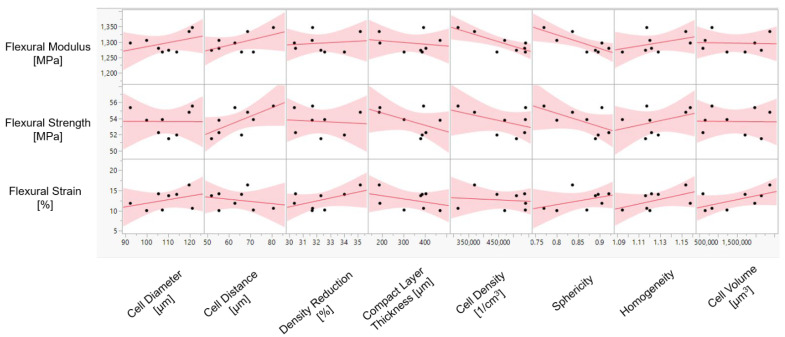
Illustration of the influence of morphological properties on mechanical characteristics of foamed polycarbonate.

Figure 5 shows the influence of the morphological properties on flexural modulus (*E_f_*), flexural strength (*σ_fM_*), and flexural strain (*ɛ_fB_*) of the foamed material. It is noticeable that there is a strong negative relationship between cell density and flexural modulus (correlation coefficient (*r*) = −0.82). The increase in sphericity of the cells (sphericity closer to 1 indicates higher cell roundness) shows a negative proportional influence on flexural modulus (*r* = −0.79). Conversely, an increase in cell diameter (median) and cell distance, and a higher variation in cell size have a positive impact on the resistance to failure. However, the strength of the relationship is relatively moderate (*r* = ≈ 0.4–0.6). Cell volume, compact layer thickness, and density reduction show a poor effect on flexural modulus compared to other morphological qualities.

**Figure 6:**
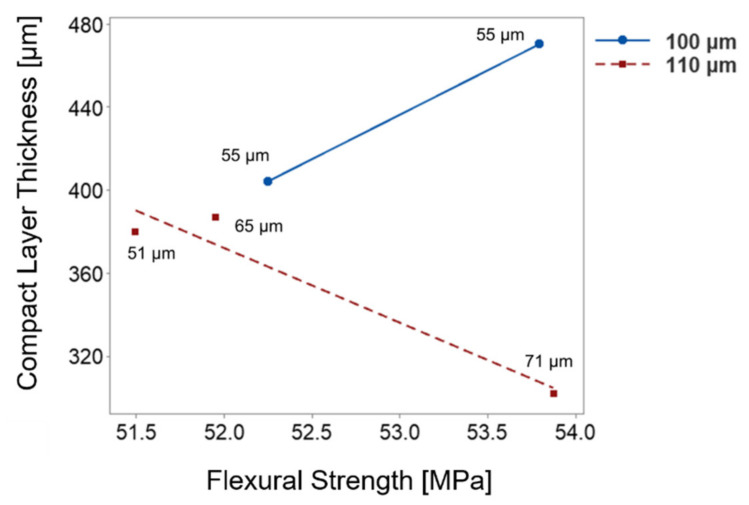
Illustration of the relationship between the flexural strength and compact layer thickness depending on cell diameter.

A further increase in average cell distance results in increasing flexural strength (*r* = 0.65). This implies that the higher amount of material concentrated along the neutral axis under loading has a positive influence on the resulting flexural strength. Because of the high interactions between the morphological properties, the relationship with flexural strength becomes ambiguous. Mechanical stiffness drops as a consequence of higher cell density. This is because cells in front of the crack act as stress concentrators [35]. Many studies have acknowledged that compact layer (skin) thickness is very important to obtain enhanced mechanical performance [5,6,23,36,37,38]. However, the results in Figure 5 show that the increased compact layer thickness has a moderate negative relationship with flexural strength (*r* = −0.50). It can be concluded that the relevant mechanical performance is not dominantly influenced by the compact layer thickness. As shown in Figure 6, the change in the thickness of the compact layer influences flexural strength depending on the cell diameter and distance between the cells. The relevant cell distance is given next to the measurement point in the figure. On the one hand, with the morphologies with the cell diameter of 100 μm, the flexural strength is enhanced with the compact layer thickness.

On the other hand, the 110 μm cell diameter dominant morphologies show the reverse relationship. The essential solid fraction is controlled with cell distance or compact skin thickness in the morphology. The change in compact layer thickness can be compensated by placing larger distances between the cells. Therefore, the impact of the compact layer on the flexural strength depends on the corresponding morphological features. In addition, toughness cannot be correlated to foam morphology, but it is noticeably affected by cell volume.

According to the best subset regression, cell distance, cell density, sphericity, and homogeneity of the morphology are identified as the best subset for the model, which significantly explains the change in material stiffness (R^2^-value = 95.52% and R^2^_adj_ value = 89.54%). The analysis of variance is given in Table 2. and the model equation is as follows:

Flexural Strength = 1.4 + 0.1403 Cell Distance + 19 × 10^−6^ Cell Density − 29.93 Sphericity + 53.3 Homogeneity (6)

**Figure 7:**
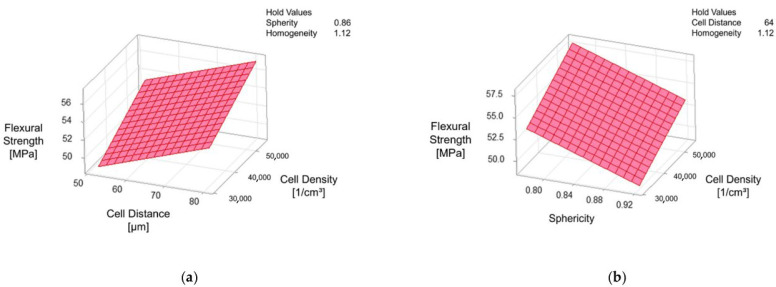
Surface plots of multiple linear regression model concerning the response of flexural strength in dependence on (**a**) cell density and cell distance, (**b**) cell density and sphericity of cells (R^2^-value 95.52%).

**Figure 8:**
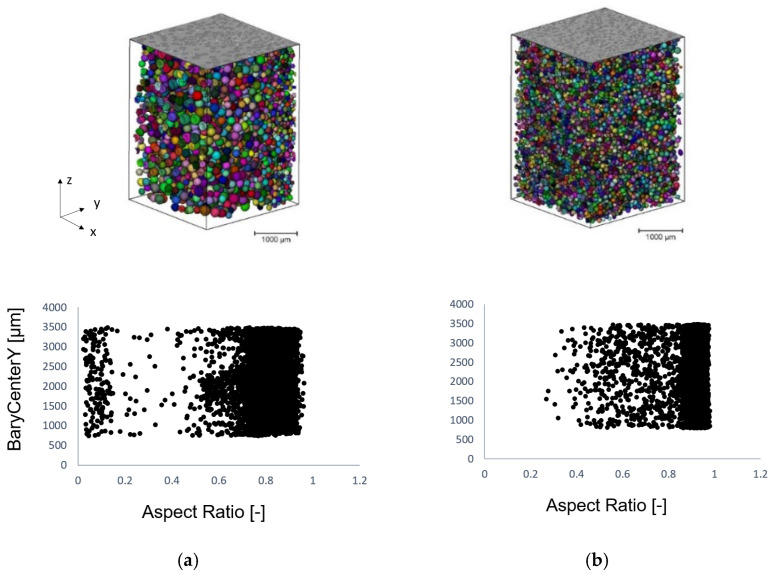
The aspect ratio depending on sample cross-section (**a**,**b**) has a cell diameter of 120 µm and 100 µm, respectively. Processing parameters of (**a**) V_in_j: 50 cm^3^/s, T_mold_: 80 °C and (**b**) V_in_j: 150 cm^3^/s, T_mold_: 30 °C, and blowing agent content of 0.4 wt% were used in the polymer melt for producing both foam structures.

The *p*-value is lower than 0.05 at 95% confidence levels presented in Table 2 and Table 3, representing statistical significance. Based on the *p*-value of the regression model given in Table 2, each included morphological explanatory variable shows a significant linear relationship depending on flexural strength. In addition, in Table 2, the degrees of freedom (DF), adjusted sum of squares (Adj SS), adjusted mean squares (Adj MS), and F-value are shown. Cell distance can explain 40% of the change in material stiffness based on the adjusted sum of squares (Adj SS) value with 4% error in the model. A homogeneity of 38%, sphericity of 34%, and cell density of 25% account for the variation in the response of flexural strength. Figure 7a reveals the interaction of the cell distance and cell density. Additionally, Figure 7b defines the coaction of the cell density and sphericity depending upon material stiffness. An increase in cell density and cell distance lead to an enhancement in flexural strength. Hong-Ru Lin determined that the cell wall thickness increases with foam density [39]. This can be attributed to an augmentation in the amount of the substantial fraction of solid that is contained in cell faces and between cells contributing to the stiffness [40]. Gibson and Ashby ascertained that, when the initial cell fluid pressure (*p*_0_) is greater than atmospheric pressure (*p_at_* = 0.1 MPa), the pressure difference *p*_0_–*p_at_* subjects the cell edges and faces in tension. By increasing this internal pressure, the collapse stress can be considerably increased. To be able to buckle, the applied stress needs to overcome the tension and buckling load of cell edges [40]. As a result, a higher cell density along with higher cell distance can be referred to escalated pressure around cell edges. During a foaming process, cell evolution occurs in the rise direction (mold cavity extension direction), which causes an anisotropic foam structure (Figure 8). The structural anisotropy in the rising direction as well as perpendicular to the rising direction leads to differences in the mechanical properties [41]. The anisotropic aspect ratio (*R*) denoted as sphericity is the ratio of cell height (*h*) (in the rising direction) to the cell width (*l*) (in the transverse direction). With respect to Figure 7b, the more cells that are elongated in expansion direction (lower sphericity), the higher the resistance arises against fracture. It can be assumed that elongated cell walls in the plane perpendicular to the load direction mainly carry the load during the deformation and inhibit the buckling of the foam specimens.

Figure 8 exemplifies the difference in the morphology in relation to the roundness of the cells. A low aspect ratio is an indicator of the elongated cells and BaryCenterY gives the position of the cells’ barycenter in the direction of “y”, which is the mold cavity expansion direction. It can be seen that the increase in cell diameter results in a higher elongation of cells throughout the cross-section of the specimens.

When it comes to the flexural modulus, the analysis of variance is given in Table 3 and the model equation is as follows:

Flexural Modulus = 808 + 0.516 Cell Diameter − 445 Sphericity + 725 Homogeneity(7)

**Figure 9:**
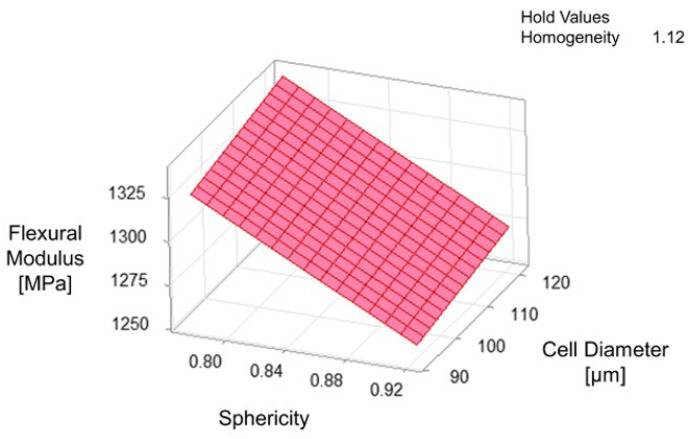
Surface plots of multiple linear regression model concerning the response of flexural modulus depending on cell diameter and sphericity of cells (R^2^-value 88.20%).

On the basis of the best subset regression model (R^2^-value = 88.20% and R^2^_adj_-value = 79.34%), cell diameter, sphericity of cells, and homogeneity of cell size distribution are found to be the best variables to predict the response of flexural modulus. It is also noticeable that sphericity has the highest Adj SS ratio in the model to explain the variation in flexural modulus. Although cell diameter has an insignificant regression coefficient, it is still included in the model to enhance the explanation of the fluctuation in flexural modulus. Figure 9 illustrates the surface plots of the model concerning flexural modulus depending on cell diameter and sphericity of cells. According to the surface plot, it is concluded that the bigger cells elongated perpendicular to the loading direction strengthen the flexural modulus. Huber and Gibson determined that the modulus ratio depends strongly on the anisotropy [33]. The loading perpendicular to the elongated cells deflects the smaller cell faces (length of *h*), resulting in increasing the stiffness. This change in the resistance to the bending can also be seen in the stress and strain diagrams (shown in Figure 4). The anisotropy leads to a stiffer and greater linear response up to the onset of the deformation of the bigger cell structures. However, a change in the sphericity of the cells can influence the flexural modulus much more than a change in cell diameter.

**Figure 10:**
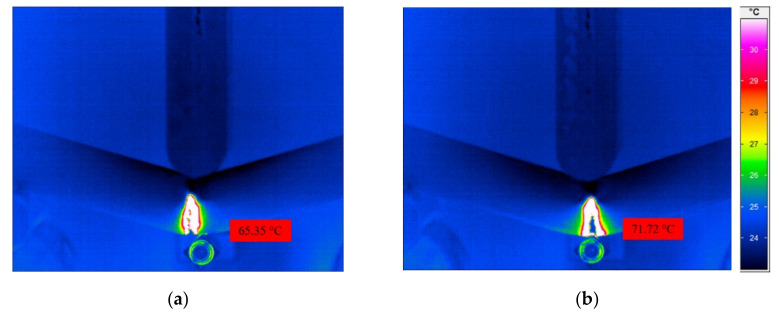

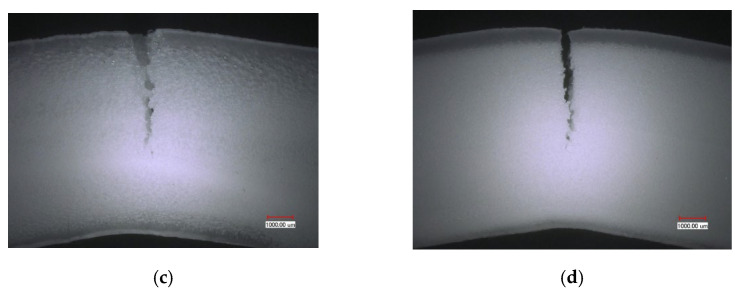
Display of the fracture temperature measurement of the specimens that have cell diameters of (**a**) 120 µm and (**b**) 100 µm, and the related appearance of the crack of the specimens: (**c**) 120 µm and (**d**) 100 µm cell diameter using digital light microscopy.

**Figure 11:**
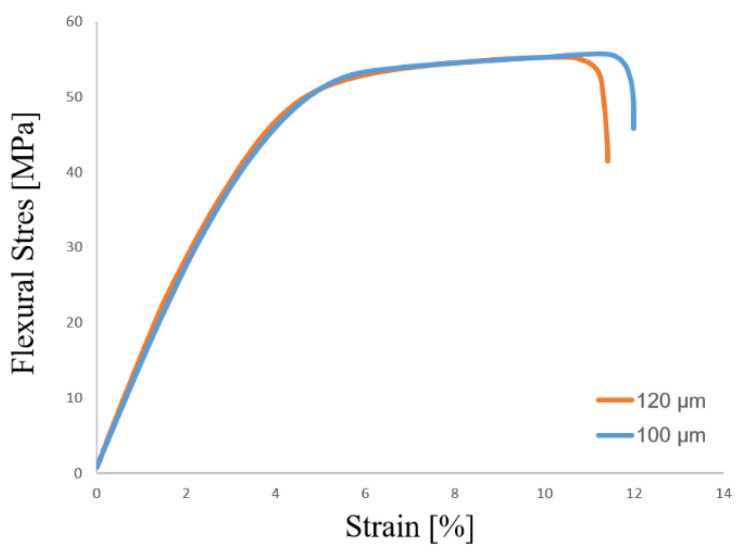
Flexural stress and strain diagram of specimens with cell diameters of 120 µm and 100 µm.

**Figure 12:**
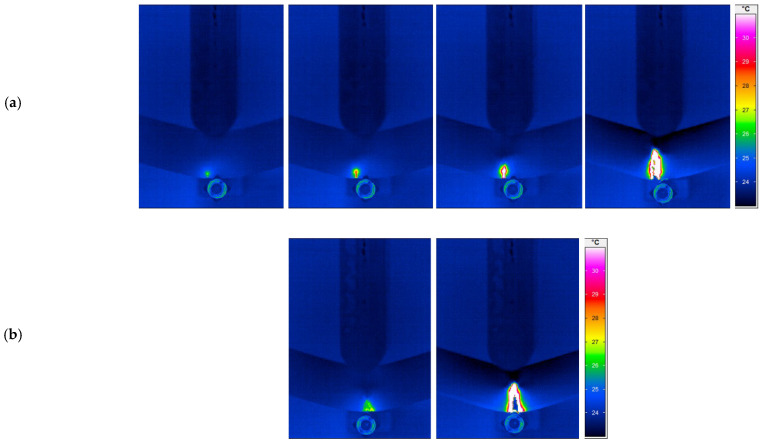
Deformation sequence during the three-point bending experiment by using an infrared thermal imaging camera: (**a**) cell diameter of 120 µm, compact layer thickness of 393.20 µm; and (**b**) cell diameter of 100 µm, compact layer thickness of 404.32 µm.

In order to establish the relationship between morphological properties and material characteristics profoundly, the fracture temperature was measured by an infrared thermal imaging camera ImageIR (IRT) (InfraTec, Dresden, Germany) during a quasi-static three-point bending test. IRBIS^®^ 3 analysis software (Infratec GmbH, Dresden, Germany) was used as a user interface for the experimental configuration as well as for the post-processing of the images. In Figure 10, the full-filled temperature distribution of the specimens with a cell diameter of (a) 120 µm and (b) 100 µm and a compact layer thickness of (a) 393.20 µm and (b) 404.32 µm is examined. Additionally, the related appearance of the crack is shown using digital light microscopy. Although the specimens acquire almost identical mechanical properties (shown in Figure 11) and the failure mode at the quasi-static bending test, the differences concerning the heat generation during the plastic deformation is more visible from infrared thermography (shown in Figure 12). Previous studies have also applied IR-thermography to study the deformation and failure mode of the cellular structures [42,43,44]. It can be seen that both cellular structures experience the bending failure in the tensile region despite the differences in cell diameter. On the one hand, the specimen with a smaller cell diameter undergoes a more sudden, brittle, as well as linear fracture (shown in Figure 10b and Figure 12b). On the other hand, a greater cell diameter accompanied with a higher cell distance results in a better interface bonding and dissipation of the generated heat in the foam structure. Therefore, the propagation of the crack is visible with a localized high heat area. Core shear failure, indentation, buckling, and plastic hinges are also presentative indicators for the deformation and failure mode (Figure 12a) [45,46].

## 4. Conclusions

In this study, the processing parameters are varied to create the distinctive foam morphologies. Thereafter, a multiple linear regression model is carried out to determine the significant structural features to predict flexural properties. According to the adopted model, cell distance, homogeneity, sphericity, and cell density are found to be the best explanatory variables to explain the change in flexural strength. Hereat, cell distance has the highest predictability, and cell density has the lowest. An increase in cell distance leads to an enhancement in stiffness, because the higher amount of material concentrated along the neutral axis contributes to the resulting flexural strength. The results signify that higher stress is necessary to propagate a crack with a larger cell structure and separation between cells. Cell diameter, sphericity of cells, and homogeneity of cell size distribution are found to be the best variables to predict the response of flexural modulus. Sphericity explains the variation in flexural modulus up to 50% and homogeneity of 23%. Cell diameter has an insignificant regression coefficient; nevertheless, it is still included in the model to enhance the predictability of the model. There is a strong negative relationship between cell density and flexural modulus, which can be attributed to the cells in front of the crack acting as stress concentrators. The bigger cells elongated perpendicular to the loading direction strengthen the flexural modulus. X-ray microtomography illustrates the difference in the morphology in relation to the roundness of the cell. The increase in cell diameter results in a higher elongation of cells throughout the cross-section of the specimens. IR-thermography enlightens the heat generation during the plastic deformation between specimens that present similar mechanical properties. The specimen with a smaller cell diameter undergoes a more sudden, brittle, as well as linear fracture. On the other hand, a greater cell diameter accompanied with a higher cell distance results in a better interface bonding and dissipation of the generated heat in the foam structure.

## Figures and Tables

**Table 1:** The variation in the processing parameters.

Parameters	Abbr.	Min	Max
Mold Temperature [°C]	T_mold_	30	80
Injection Flow Rate [cm^3^/s]	V_inj_	80	150
Blowing Agent Content [wt%]	SCF-Level	0.4	0.8

**Table 2:** Regression analysis of variance for the prediction of flexural strength of physically foamed polycarbonate.

Source	DF	Adj SS	Adj MS	F-Value	*p*-Value
Regression	4	16.67	4.16	15.98	0.023
Cell Distance [μm]	1	7.02	7.02	26.94	0.014
Cell Density [1/cm^3^]	1	4.43	4.43	17.01	0.026
Sphericity	1	5.95	5.95	22.81	0.017
Homogeneity	1	6.67	6.67	25.58	0.015
Error	3	0.78	0.26		
Total	7	17.45			

DF indicates degrees of freedom; Adj SS, adjusted sums of squares; Adj MS, adjusted mean squares.

**Table 3:** Regression analysis of variance for the prediction of flexural modulus of physically foamed polycarbonate.

Source	DF	Adj SS	Adj MS	F-Value	*p*-Value
Regression	3	5775.2	1925.1	9.96	0.025
Cell Diameter [μm]	1	143.5	143.5	0.74	0.437
Sphericity	1	3258.3	3258.3	16.86	0.015
Homogeneity	1	1537.8	1537.8	7.96	0.048
Error	4	773.0	193.2		
Total	7	6548.2			

DF indicates degrees of freedom; Adj SS, adjusted sums of squares; Adj MS, adjusted mean squares.
